# Physiological and Gene Expression Changes of *Clematis crassifolia* and *Clematis cadmia* in Response to Heat Stress

**DOI:** 10.3389/fpls.2021.624875

**Published:** 2021-03-26

**Authors:** Qingdi Hu, Renjuan Qian, Yanjun Zhang, Xule Zhang, Xiaohua Ma, Jian Zheng

**Affiliations:** ^1^Zhejiang Institute of Subtropical Crops, Wenzhou, China; ^2^China National Bamboo Research Center, Hangzhou, China

**Keywords:** *Clematis crassifolia*, *Clematis cadmia*, heat stress, physiological analysis, gene expression

## Abstract

*Clematis* is a superior perennial ornamental vine known for varied colors and shapes of its flowers. *Clematis crassifolia* is sensitive to high temperature, whereas *Clematis cadmia* has a certain temperature adaptability. Here we analyzed the potential regulatory mechanisms of *C. crassifolia* and *C. cadmia* in response to heat stress by studying the photosynthesis, antioxidant parameters, amino acids, and gene expression patterns under three temperature treatments. Heat stress caused the fading of leaves; decreased net photosynthetic rate, stomatal conductance, superoxide dismutase, and catalase activity; increased 13 kinds of amino acids content; and up-regulated the expression of seven genes, including C194329_G3, C194434_G1, and C188817_g1, etc., in *C. crassifolia* plants. Under the treatments of heat stress, the leaf tips of *C. cadmia* were wilted, and the net photosynthetic rate and soluble protein content decreased, with the increase of 12 amino acids content and the expression of c194329_g3, c194434_g1, and c195983_g1. Our results showed that *C. crassifolia* and *C. cadmia* had different physiological and molecular response mechanisms to heat stress during the ecological adaptation.

## Introduction

*Clematis* is a large genus that belongs to Ranunculaceae and has great ornamental value. There are various species of *Clematis*, which have rich variations in colors and shapes of flower. *Clematis* can be widely used in flower stands, corridors, lamp pillars, fences, arches, and other configurations to form an independent landscape, and it occupies a very important position in landscaping (Lehtonen et al., 2016). *Clematis* also has a certain medicinal value, which can be used as anti-inflammatory, antirheumatism, and analgesic agents (Hao et al., 2013; Li et al., 2017). There are approximately 355 species of *Clematis* in the world and 147 species in China (Pringle, 1971; Liu et al., 2018). In terms of adaptability to the environment, *Clematis* prefers a cool climate and is often associated with shrubs in the wild. At present, most cultivars of *Clematis* on the market are susceptible to high temperature in summer (Gao et al., 2017; Jiang et al., 2020). After the heat stress, the leaves will fade, wilt, and fall off, stem will wither, and there will be other heat damage symptoms, which severely affects the ornamental characteristics of *Clematis*.

With the global warming caused by the greenhouse effect, the heat stress has become one of the most important abiotic stresses that restrict plant growth (Berry and Bjorkman, 1980). In recent years, the frequent occurrence of extreme high temperature posed a severe challenge to the ability of plants to withstand high temperatures (Hasanuzzaman et al., 2013; Borrell et al., 2020). Therefore, the regulatory mechanism of plants in response to heat stress and the cultivation of heat-resistant varieties have become a focus of attention. Plants have produced a series of adaptation mechanisms to resist heat stress in the process of evolution (Wahid et al., 2007). One is heat resistance, which strengthens plants by changing leaf orientation, increasing leaf trichomes, and increasing xylem cells (Tozzi et al., 2013; Bickford, 2016). The other is heat tolerance mechanism, which is involved in a series of signal pathways, including ion transporters, osmoprotectants, free radical scavengers, signal cascades, and transcription factor regulation (Rodríguez et al., 2008; Ohama et al., 2017).

Previous studies have shown that heat stress changes the components and structure of plant cell membranes, reduces cell membrane integrity, increases cell membrane permeability, and causes the ion leakage (Wise et al., 2004; Bita and Gerats, 2013; Lohani et al., 2020). Heat stress can cause plants to accumulate excess reactive oxygen species (ROS), break the balance of ROS in cells, inhibit the photosynthetic electron transport chain, and cause irreversible damage to photoresponse system II while intensifying membrane lipid peroxidation (Ahmad et al., 2010; Gururani et al., 2015; Choudhury et al., 2017). Plants have evolved both enzymatic and nonenzymatic systems to remove ROS to maintain the growth of plants. The currently reported enzymatic antioxidant systems include superoxide dismutase (SOD), catalase (CAT), and ascorbic peroxidase (APX), etc. Nonenzymatic antioxidants are antioxidants in plants such as proline, ascorbic acid, mannitol, and glutathione, etc (Hameed et al., 2012; Tripathy and Oelmüller, 2012). In *Lilium longiflorum*, the antioxidant enzyme activities including SOD, peroxidase (POD), CAT, APX, and glutathione reductase (GR) were stimulated after 10 h of high-temperature treatment at 37°C and 42°C, and the concentrations of ascorbic acid (AsA) and glutathione (GSH) were maintained at a high level, resulting in the decrease of ROS content, so as to mitigate the damage caused by heat stress (Yin et al., 2008). The accumulation of osmotic regulation substances is also an important physiological mechanism for plants to respond to heat stress (Kaplan et al., 2004).

The up-regulated expression of genes has been reported to help plants adapt to heat stress (Atkinson and Urwin, 2012). Heat shock proteins (HSPs) are a type of stress protein that is induced in organisms under the heat stress (Jacob et al., 2017). They are involved in protein synthesis, folding, cell localization, protein transmembrane transport, and target protein degradation to maintain the stability of the plant homeostasis (Qu et al., 2013; Xu et al., 2016). Heat shock transcription factors (HSFs) are the key regulator of plant response to heat stress and play a critical role in the regulation of plant heat stress response (Guo et al., 2016). HsfA1 was an important transcription factor for *Arabidopsis thaliana* to obtain heat resistance (Ohama et al., 2016). The transcription factor FaHsfA2c of *Festuca arundinacea* was upregulated in leaves and roots under heat stress, which could enhance the heat resistance of *F. arundinacea* (Wang et al., 2017). In tomato (*Solanum lycopersicum*), Mishra founded that plants with silenced of *HsfA1a* were more sensitive to heat stress than wild type in each developmental stage (Mishra et al., 2002). HsfB1 was a transcriptional inhibitor, and it could also be used as coactivator of HsfA1a to inhibit the expression of *HsfA1b* and *HsfA2* (Röth et al., 2017; Zhou et al., 2018).

As an excellent ornamental vine, the market demand for *Clematis* is constantly rising in the world. However, the continuous loss of wild resources and the limited heat tolerance of horticultural varieties put forward a severe test to the cultivation of *Clematis*. How to effectively improve the heat resistance and reduce the damage of heat stress is the emphasis work of cultivation and breeding of *Clematis*. *Clematis crassifolia* is a kind of evergreen species, which blooms in winter and is mostly distributed in dense forests or sparse forests with an altitude of 100–300 m in China. *Clematis cadmia* is a potential material for resistance breeding because of its strong resistance and abundant flowers. In preliminary study, we found that *C. cadmia* has a certain temperature adaptability, whereas *C. crassifolia* was more sensitive to temperature in summer, so it is necessary to analyze the physiological and biochemical differences between *C. crassifolia* and *C. cadmia*. In this study, in order to understand the effects of heat stress on *Clematis*, we explored the response of photosynthesis, antioxidant enzyme activity system, amino acid levels, and gene expression in *C. crassifolia* and *C. cadmia*, which is expected to provide a theoretical foundation for the cultivation and breeding of *C. crassifolia* and *C. cadmia*.

## Materials and Methods

### Plant Materials and Growth Conditions

Plants of *C. crassifolia* and *C. cadmia* were grown under different heat stress and conducted in the Zhejiang Institute of Subtropical Crops (120°37′53″E, 28°0′8″N), China. Two-year-old healthy and homogenous plants were grown in a grown chamber under a 16/8-h long-day cycle at 25°C/20°C, 65% humidity for 2 weeks. After 2 weeks of pretreatment, in order to carry out the heat stress, the pretreated plants of *C. crassifolia* and *C. cadmia* were transferred to the grown chamber for cultivation at 25°C/20°C, 35°C/30°C, and 45°C/40°C temperature, respectively. Heat stress treatments lasts for 4 days; during the treatment period, water was poured every 2 days, 500 mL each time, to ensure sufficient soil moisture. Take the mature leaves of two to five positions after 4 days of treatments, respectively. Fresh leaves were tested for physiological indicators, and other samples were frozen in liquid nitrogen and stored at −80°C for the analysis of amino acid and gene expression. Experimental treatments were repeated three times.

### Leaf Gas Exchange Parameters

Healthy and fully developed leaves from each treatment were randomly chosen for photosynthetic parameter measurements, using LI-6400 XT portable photosynthesis system (Li-Cor Inc., Lincoln, NE, United States), and equipped with a 6400-18 RGB LED light source. The measurements were carried out from 9:00 to 11:00 AM, the photosynthetic photon flux density was 1200 μmol m^–2^ s^–1^, the CO_2_ concentration was 400 ppm, and the relative humidity was 65%.

### Photosynthetic Pigments

The finely cut and well-mixed leaf samples (100 mg) were transferred to a 10 mL tube. Then, 8 mL of 80% acetone was added to the test tube and mixed. Chlorophyll was extracted at 4°C in the dark. The absorbance of supernatant was measured at 663, 645, and 470 nm with a spectrophotometer (Shimadzu UV-2550, Kyoto, Japan). The total chlorophyll content was calculated and was expressed as mg g^–1^ FW. The total chlorophyll content was measured according to the described method by Lichtenthaler and Buschmann (2001).

### Measurement of MDA Content, Hydrogen Peroxide Content, and Soluble Protein Content

The malondialdehyde (MDA) content was determined as previously described (Ouyang et al., 2010).

The H_2_O_2_ content and soluble protein content were measured according to the method as previously described (Luo et al., 2012).

### Determination of SOD, CAT, and POD Activity

For antioxidant enzyme activity analysis, fresh leaves (0.1 g) were ground in liquid nitrogen and then suspended in 8.0 mL solution containing 50 mM phosphate buffer (pH 7.4). The homogenate was centrifuged 10,000 rpm for 15 min at 4°C, and the supernatant was collected to obtain crude enzymes.

The SOD activity was analyzed by measuring the inhibiting rate of the enzyme to O_2_^–^ produced. One-unit SOD activity (U) was defined as the amount of enzyme that resulted in 50% inhibition of reduction of nitrite in 1 mL reaction solution. The SOD activity was determined at 550 nm after 40 min of reaction at 37°C and expressed as U g^–1^ FW (Ma et al., 2017).

The CAT activity was determined by the hydrolysis reaction of hydrogen peroxide (H_2_O_2_) with CAT; the reaction could be terminated rapidly by molybdenum acid (MA) to produce yellow MA-H_2_O_2_ complex (Li et al., 2013). The CAT activity was calculated in absorbance at 405 nm. One unit was defined as the amount of enzyme that resulted in the decompose of 1 μmol H_2_O_2_ per second in 1.0 g fresh tissue.

The POD activity was measured based on the change of absorbance at 470 nm by catalyzing H_2_O_2_ (Zheng et al., 2018). One unit was defined as the amount of enzyme that resulted in the change of 0.01 at 470 nm per minute by 1.0 g fresh tissues in the reaction system.

### Amino Acid Contents Analysis

Samples (10 mg) were weighed, mixed with 1 mL methanol, and subsequently homogenized in an ultrasonic instrument for 3 min, tubes were static for 5 min at room temperature, and 10,000 rpm centrifuged for 15 min at 4°C. The supernatant was diluted 10 times. One hundred microliters of dilution was transferred to heat-resistant tubes, and 100 μL of internal standard solution (100 ppb) was added; the mixture was filtered through a 0.45-μm membrane and then injected into the Ultra Performance Liquid Chromatography (UPLC) for analysis. The 24 kinds of standards were weighed accurately. Stock solutions were prepared using methanol or water, and a series of mixed working standard solutions were properly prepared and diluted with water. The standard solutions were stocked under 0°C.

UPLC separation was performed on an Acquity UPLC system (Waters, United Kingdom) equipped with an ACQUITY UPLC^®^ BEH HILIC (1.7 μm, 2.1 × 100 mm, Waters) column. The temperature of the column was set at 40°C. The sample injection volume was 5 μL. Eluents consisted in water/methanol (90:10) with 0.1% (vol/vol) formic acid (eluent A) and water/methanol (50:50) with 0.1% (vol/vol) formic acid (eluent B). The gradient elution started with 10% B for 0 min, ramped to 30% of B within next 6.5 min, ramped to 100 % of B in 7 min, kept at 100% of B until 8 min and dropped to 10% B in 8.5 min at a flow rate of 0.3 mL/min, and finally kept at 10% of B until 12.5 min at a flow rate of 0.4 mL/min.

The MS analysis was performed using a AB 4000 mass spectrometer (AB, United States) equipped with an ESI source in the positive-ion mode working in the multiple reaction monitoring mode. An ion source voltage of 5.5 kV and a source temperature of 500°C were used. Collision gas and the curtain gas were set at 6 and 30 psi, respectively, whereas both of the atomization gas and auxiliary gas were 50 psi.

### Gene Expression

Isolation of total RNA from the leaf tissues and real-time quantification of transcriptional expression of the genes were the same as that reported previously (Ma et al., 2019). All of the primers used for quantitative reverse transcriptase–polymerase chain reaction (PCR) are listed in Supplementary Tables 1, 2. All of the PCR products were confirmed by sequencing.

### Statistical Analysis

Data were analyzed by two-way analysis of variance (ANOVA) using the SPSS 10 program (SPSS Inc., Chicago, IL, United States). Different letters on the histograms between different treatments indicate their statistical difference at *P* ≤ 0.05.

## Results

### High-Temperature Stress Caused Leaf Discoloration and Crimping of *C. crassifolia* and *C. cadmia*

**FIGURE 1:**
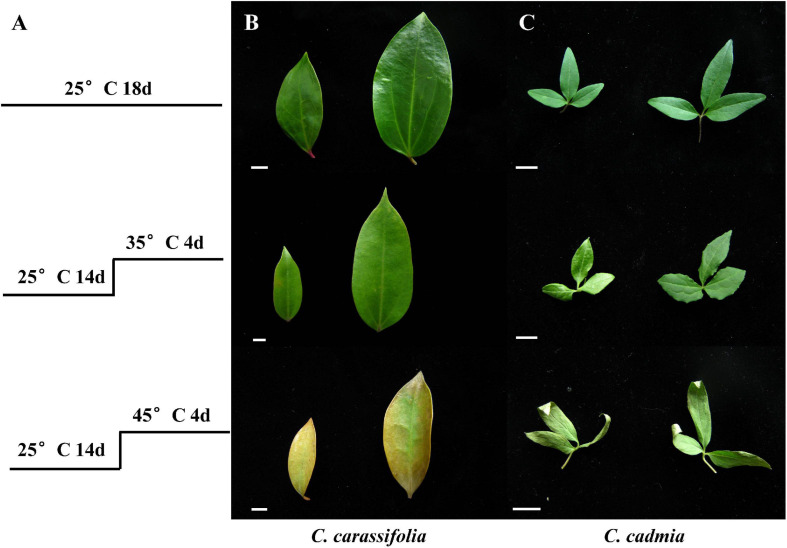
Effect of heat stress on the morphology of the leaves of *C. crassifolia* and *C. cadmia*. **(A)** Treatment conditions. **(B)** The phenotype of *C. crassifolia* leaves. **(C)** The phenotype of *C. cadmia* leaves. Bar means 1 cm.

Under different temperature treatments, the leaves damage degree of *C. crassifolia* and *C. cadmia* showed significant differences. In comparison with the normal-temperature treatment (25°C), *C. crassifolia* mainly showed water loss, leaf carnification degree reduced, and leaf turned yellow (Figure 1B). *C. cadmia* exhibited partial curling with the yellow leaf tips (Figure 1C). In addition, after 45°C heat stress treatment, the MDA content of *C. crassifolia* was increased 443.97% compared with normal-temperature treatment (25°C). No significant differences were observed in *C. cadmia* between two heat stress treatments (Supplementary Figure 1).

### Heat Stress Inhibited the Photosynthesis of *C. crassifolia* and *C. cadmia*

**FIGURE 2:**
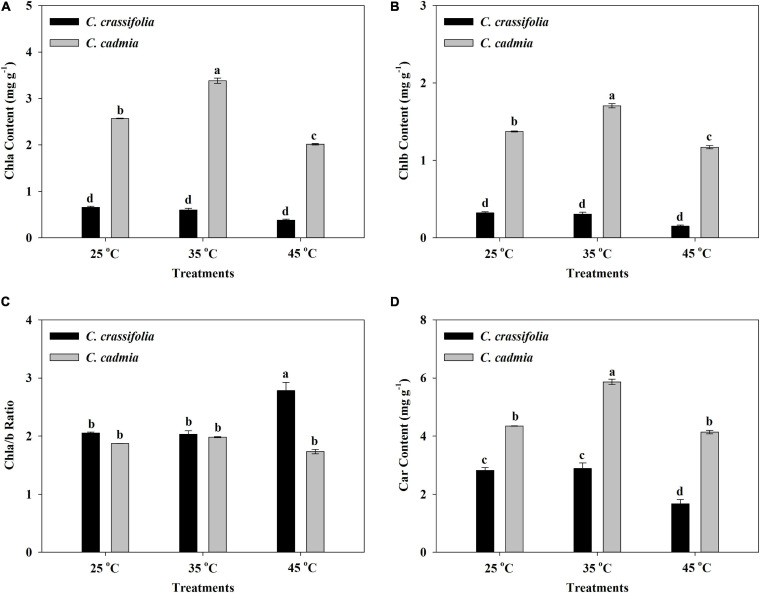
Chlorophyll a (Chla), chlorophyll b (Chlb), chlorophyll a/b ratio, and carotenoid (Car) in *C. crassifolia* and *C. cadmia* grown under three different temperatures including normal temperature (25°C), moderate temperature (35°C), and high temperature (45°C). **(A)** Chla content. **(B)** Chlb content. **(C)** Chla/b ratio. **(D)** Car content. Error bars indicate SE (*n* = 5 plants). Different letters indicate significant differences based on two-way ANOVA followed by Tukey multiple comparisons (*P* ≤ 0.05).

The heat stress can aggravate the degradation of chlorophyll and inhibit its synthesis, so the change of chlorophyll content can reflect the damage degree of high temperature to plants (Zhou and Leul, 1999). According to the heat stress treatments, the Chla, Chlb, and carotenoid content of *C. crassifolia* were decreased, instead of the increased Chla/b ratio. Even at the same heat stress condition, *C. cadmia* plants have high photosynthetic pigments than *C. crassifolia*; the Chla and Chlb content under the moderate (35°C) temperature increased by 31.59% and 24.10%, respectively, compared with those under the normal temperature (25°C) in *C. cadmia*, and decreased by 21.59% and 14.79% under high (45°C) temperature compared with the normal temperature (25°C), respectively (Figure 2).

**FIGURE 3:**
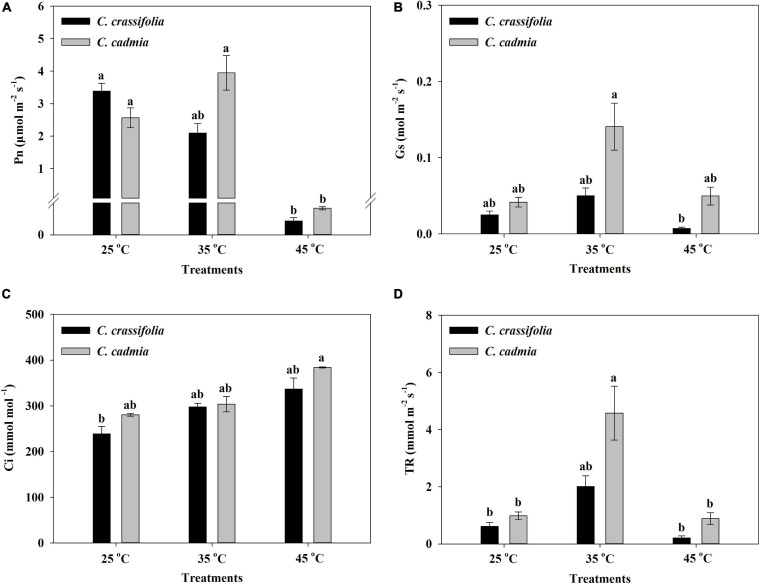
Photosynthetic parameters of *C. crassifolia* and *C. cadmia* grown under three different temperatures including normal temperature (25°C), moderate temperature (35°C), and high temperature (45°C). **(A)** Net photosynthetic rate (P_n_). **(B)** Stomatal conductance (Gs). **(C)** Intercellular CO_2_ concentration (C_i_). **(D)** Transpiration rate (TR). Error bars indicate SE (*n* = 5 plants). Different letters indicate significant differences based on two-way ANOVA followed by Tukey multiple comparisons (*P* ≤ 0.05).

The photosynthetic parameters of *C. crassifolia* and *C. cadmia* were also strikingly affected by heat stress treatments. The net photosynthetic rate (P_n_) of *C. crassifolia* and *C. cadmia* showed significant decrease in high-temperature treatment (45°C), which were only 1.29% and 3.25% of normal-temperature treatment (25°C), respectively (Figure 3A). With the increase of temperature, the variation trends of stomatal conductance (Gs), intercellular CO_2_ concentration (Ci), and transpiration rate (TR) of *C. crassifolia* and *C. cadmia* were similar. *C. crassifolia* plants grown under 45°C condition have extremely low Gs and TR values, decreased 71.58% and 65.80%, respectively, compared with the plants under normal temperature (25°C). However, the Gs, Ci, and TR of *C. cadmia* has no significant difference between 25°C and 45°C treatments (Figures 3B–D).

### The Antioxidant System of *C. crassifolia* Was More Influential Than *C. cadmia* Under Heat Stress

**FIGURE 4:**
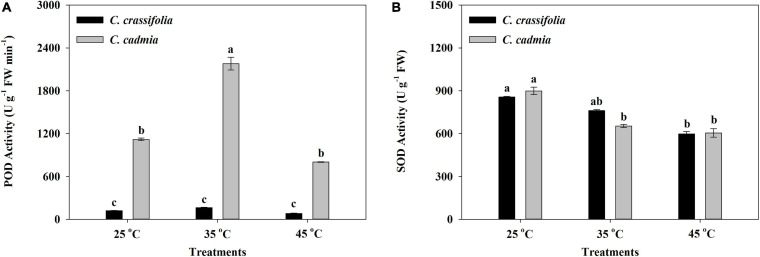
Effect of heat stress on the peroxidase (POD) activity and the superoxide dismutase (SOD) activity of *C. crassifolia* and *C. cadmia* grown under three different temperatures. **(A)** POD activity. **(B)** SOD activity. Error bars indicate SE (*n* = 5 plants). Different letters indicate significant differences based on two-way ANOVA followed by Tukey multiple comparisons (*P* ≤ 0.05).

The activities of POD and SOD in *C. cadmia* leaves were significantly affected by heat stress treatments. The POD activity was 1.95 times that in the normal-temperature treatment (25°C). The SOD activity was decreased significantly with different heat stress, by 27.33% and 32.79%, respectively. In *C. crassifolia*, there was no significant difference of POD activity among three temperature degrees, but SOD activity decreased gradually with the increase of temperature (Figure 4).

**FIGURE 5:**
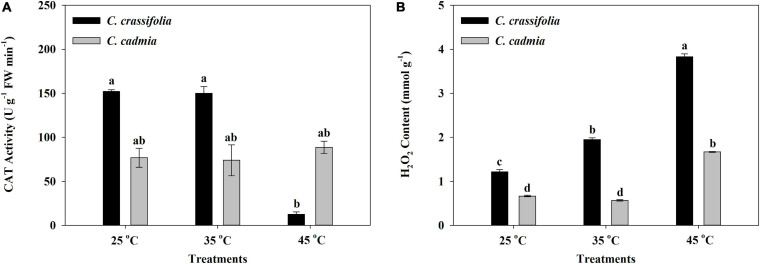
Effect of heat stress on the Catalase (CAT) activity and the peroxide (H_2_O_2_) content of *C. crassifolia* and *C. cadmia* grown under different temperatures. **(A)** CAT activity. **(B)** H_2_O_2_ content. Error bars indicate SE (*n* = 5 plants). Different letters indicate significant differences based on two-way ANOVA followed by Tukey multiple comparisons (*P* ≤ 0.05).

The CAT activity of *C. crassifolia* was significantly inhibited by heat stress. Under the condition of 45°C heat stress, the activity of CAT decreased by 91.76%, whereas there was no significant change in *C. cadmia* plants (Figure 5A), while the H_2_O_2_ content in *C. crassifolia* was increased obviously by gradient with the increase of temperature, increased by 60.64% and 215.54%, respectively, under moderate (35°C) and high (45°C) temperature compared with the normal temperature (25°C). The *C. cadmia* plants exposed to high-temperature treatment (45°C) showed 150.60% increase of the H_2_O_2_ content than those under normal (25°C) treatment (Figure 5B).

### The Content of Soluble Protein and Amino Acid in *C. crassifolia* and *C. cadmia* Showed Difference Patterns Under Heat Stress

**FIGURE 6:**
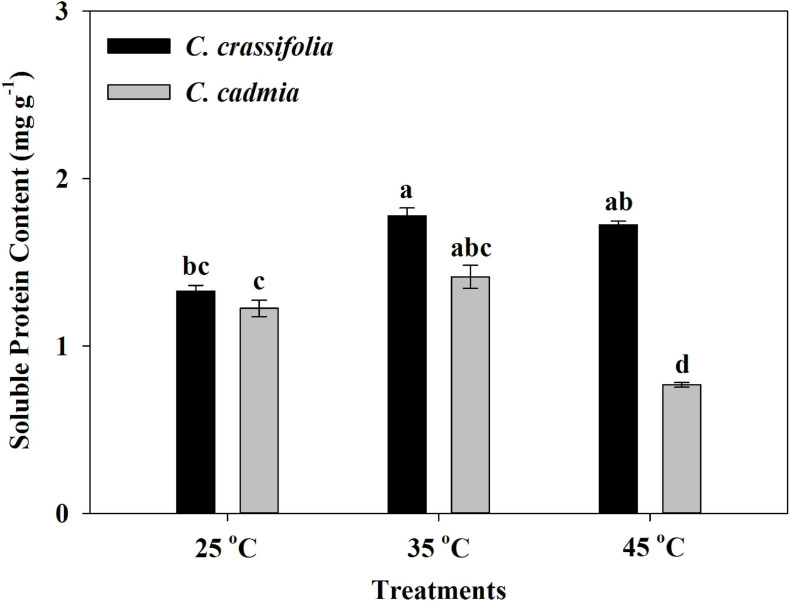
Soluble protein content of *C. crassifolia* and *C. cadmia* grown under three different temperatures. Values are the means ± standard error (*n* = 5 plants). Different letters indicate significant differences based on two-way ANOVA followed by Tukey multiple comparisons (*P* ≤ 0.05).

After different heat treatments, the soluble protein content of *C. cadmia* decreased 45.67% under high-temperature treatment (45°C) compared with normal-temperature treatment (25°C); however, there was no significant change of soluble protein content in *C. crassifolia* plants under heat-stressed conditions (Figure 6).

The amino acid content was assessed in *C. crassifolia* and *C. cadmia* plants under different heat stress treatments (Table 1). Isoleucine, glutamine, histidine, and tryptophan were significantly increased in both *C. crassifolia* and *C. cadmia* plants under moderate-temperature (35°C) and high-temperature (45°C) treatments compared with normal-temperature treatment (25°C). Ornithine hydrochloride was only detected in *C. crassifolia* plants, and glycine was only detected in *C. cadmia* under 45°C heat treatment. Proline, valine, threonine, lysine, phenylalanine, and tyrosine were up-regulated at 45°C condition in *C. crassifolia* and at 35°C treatment in *C. cadmia* (Table 1).

**TABLE 1:**
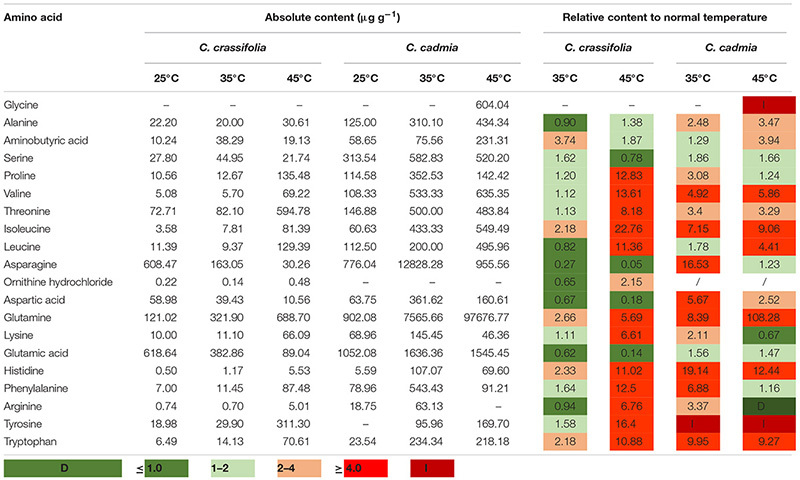
Amino acid contents (data shown are the mean) of *C. crassifolia* and *C. cadmia grown* under different temperature treatments.

*D, detected in normal temperature but not in moderate- and high-temperature treatments; I, detected in moderate and high temperature but not detected in normal-temperature treatments; —, indicates not detectable.*

### Gene Expression Pattern in *C. crassifolia* and *C. cadmia* Under Heat Stress

**FIGURE 7:**
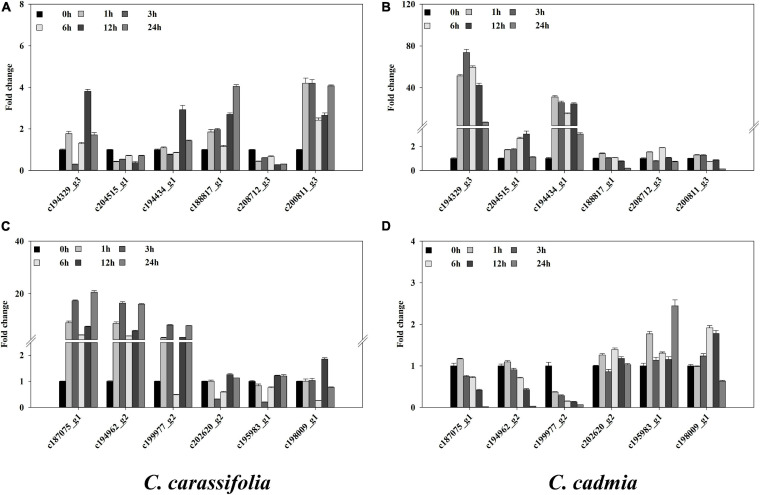
Effect of heat stress on the gene expression in *C. crassifolia* and *C. cadmia* leaves. Time course of heat in *C. crassifolia* and *C. cadmia* shifted to 45°C. **(A,C)** *C. carassifolia*. **(B,D)** *C. cadmia*. Soluble protein content. Bars indicate SE (*n* = 3).

To explain the response of *C. crassifolia* and *C. cadmia* plants to heat stress, we examined the expression of 12 genes under 45°C culture condition, including HSP, HSF, photosystem, and POD genes, which were descripted in transcriptome profiling (Figure 7). The transcript levels were significantly different in *C. crassifolia* and *C. cadmia.* The expression levels of HSP and HSF genes including c194329_g3 and c194434_g1 in *C. cadmia* were remarkably higher (>2.0-fold) after heat stress, and those two genes have significant up-regulation in *C. crassifolia* except endure the heat stress for 12 h (Figures 7A,B). Four genes associated with heat stress, including c188817_g1, c200811_g3, c187075_g1, and c194962_g2, which were involved in biosynthesis of photosystem and chlorophyll, have high transcript levels after heat stress in *C. crassifolia* plants, and only c188817_g1 and c208712_g3 showed a small amount of increase after 6 h of heat stress treatment in *C. cadmia*. For antioxidant enzyme genes, the transcript level of c199977_g2 has substantially increased in *C. crassifolia* after heat stress, and the expression of c202620_g2, c195983_g1 and c198009_g1 varied slightly in *C. cadmia* (Figures 7C,D).

## Discussion

With the aggravation of greenhouse effect and the rise of global temperature, heat stress is the main environmental stress that restricts plant growth and development. Plants respond to heat stress through changes in physiological, biochemical, and transcriptional regulatory systems (Baniwal et al., 2004; Rizhsky et al., 2004; Wu et al., 2018). This study presents the leaves phenotype, physiological mechanism, and gene expression pattern of *C. crassifolia* and *C. cadmia* in response to different heat stress. The damage to plant organs, tissues, and cells caused by heat stress was multifold. Plants exposed to heat stress damage showed discoloration or peeling of leaves, damage of flowers and fruits, poor seed germination rate, and inhibition of plant growth (Rodríguez et al., 2005). With the increase of temperature, *C. crassifolia* and *C. cadmia* manifested different symptoms of heat damage. The fading and wilting leaves were observed in *C. crassifolia*, whereas *C. cadmia* leaves showed rolling and drying in leaf tip and leaf margins (Figure 1).

Photosensitive pigment regulation is an important regulation mechanism of photosynthesis protection in plants under heat stress. Increasing the chlorophyll content with a certain range can improve the absorption and transformation of light energy by plants (Shi et al., 2006). In this study, the content of Chla and Chlb decreased under the 45°C heat stress and caused a 35.49% increase in Chla/b and a 40.84% decrease in *C. crassifolia* (Figures 2A–C), indicating that Chlb was more sensitive to heat stress than Chla in *C. crassifolia*. Heat stress affects the early stage of photosynthesis and mainly changes the membrane properties of chloroplasts and the uncoupling of energy transfer mechanism, but protein denaturation under continuous heat stress can cause irreversible damage (Fan et al., 2018). The reasons for the decrease of Pn in plant leaves are mainly divided into stomata limiting factors and non–stomata-limiting factors. The previous studies have observed that the heat stress can cause plant stomatal closure and reduce the photosynthetic rate (Crafts-Brandner and Salvucci, 2002; Pnueli et al., 2003).

In this study, the Pn, Gs, and Tr of *C. crassifolia* were significantly reduced under heat stress of 45°C, indicating that heat stress caused the decline in photosynthesis of *C. crassifolia* by both stomata-limiting factors and non–stomata-limiting factors. The total chlorophyll content of *C. cadmia* decreased significantly at 45°C heat stress, and there were no significant differences in Gs and Tr, whereas the increased of Gs and Tr were observed in *C. cadmia* plants under 35°C, resulting in a 54.02% increase in Pn. These results indicated that *C. cadmia* can adapt to the environment by increasing the chlorophyll content and Gs under moderate temperature conditions, and the decrease of photosynthetic activity under heat stress was the main reason for the reduction of photosynthesis.

The injury of plants under adversity is closely related to membrane lipid peroxidation induced by the accumulation of ROS (Asada, 2006). MDA is one of the most important products of membrane lipid peroxidation. Therefore, MDA level can be measured as an indirect measure of injury to membrane lipid and stress resistance of plants. In this study, we observed that 45°C heat stress led to a significant increase in MDA content and H_2_O_2_ content of *C. crassifolia*; however, there were no significant changes in *C. cadmia* under different temperature treatments (Supplementary Figure 1 and Figure 5B). The results suggested that the high temperature could result in the accumulation of excessive H_2_O_2_ in *C. crassifolia*, which would cause the destruction of cell membrane. But *C. cadmia* could maintain a relatively stable cell homeostasis under heat stress.

Plant in long-term evolution formed in the process of enzymatic reaction system in order to eliminate the oxidative stress caused by heat stress and enhance the protection ability of plants, including POD, CAT, and SOD, etc., which play a key role in the regulation of ROS homeostasis in the cell (Larkindale and Vierling, 2008; Vidya et al., 2018). The stress tolerance of tomatoes at heat is closely associated with its antioxidant mechanism (Zhou et al., 2019). In our study, it was shown that *C. crassifolia* and *C. cadmia* could respond to heat stress by regulating the antioxidant mechanism, but sustained heat stress would reduce the enzyme activity.

Amino acids are a class of important physiological active substances. Substances such as amino acids or polyamines synthesized with amino acids as precursors can accumulate under heat stress, stabilize proteins, and maintain cell osmotic pressure (Bowlus and Somero, 1979). Glycine, as an important amino acid, is a synthetic substrate of glycine betaine (GB), which can protect photosystem II, stabilize membranes, and reduce oxidative damage (Sita et al., 2018; Alhaithloul et al., 2020). γ-Aminobutyric acid (GABA) is a nonprotein amino acid widely present in plants. Under heat stress, GABA can improve the activity of antioxidant enzymes such as POD and CAT to reduce peroxidation damage (Nayyar et al., 2014). Exogenous application of GABA could enhance accumulation of osmolytes such as proline and trehalose due to increase in the activities of their biosynthetic enzymes and improved the leaf turgor, carbon fixation, and assimilation processes to protect the reproductive function from heat stress in mungbean (Priya et al., 2019). In this study, amino acids such as Pro, Val, Thr, Ile, Leu, Glu, Lys, His, and Tyr were increased to relieve osmotic pressure of leaf cell in *C. crassifolia*. *C. cadmia* sufficiently increased the content of amino acids such as Gly, GABA, Glu, and Tyr; maintained the activity of antioxidant enzymes; and reduced the content of MDA, thereby enhancing the stability of cell membrane structure and alleviating the damage caused by heat stress.

Plant response to heat stress involves a complex gene regulatory network, and the damage caused by stress can be alleviated by regulating the expression of related genes (Krasensky and Jonak, 2012). In the previous study, a total of 81 SRAP and 133 EST-SSR polymorphic loci were detected in *Clematis* (Li et al., 2018). HSPs, especially small HSPs, antioxidant enzymes (e.g., APX), and galactosyl alcohol synthesis enzymes play key roles in the heat resistance of grapes (Liu et al., 2012). The HSF HsfA1 plays an important role in transcriptional regulatory networks in promoting the expression of heat stress–related genes, to regulate intracellular protein activity, rehabilitate denatured proteins, degrade misfolded proteins, and alleviate the damage caused by heat stress (Yoshida et al., 2011). We screened out the expression patterns of 12 genes associated with heat stress (Figure 7). The expressions of c194329_g3, c204515_g1, c194434_g1, and c195983_g1 were up-regulated in *C. cadmia* within a short period of heat stress, whereas the response time of *C. crassifolia* was longer. c194329_g3 and c194434_g1 were up-regulated 12 h later. The results showed that *C. cadmia* could rapidly respond to heat shock stress and promote the synthesis of related enzymes and metabolites by enhancing the expression of small HSP, HSFs, and APX genes, thus alleviating the damage caused by heat stress. PsaH is a membrane peripheral protein located at the surface of PSI, and PSBY protein is located in the thylakoid membrane, both of which play an important role in photosynthetic system composition and electron transport (Obokata et al., 1993; Ozawa et al., 2018). *C. crassifolia* leaves photosynthetic system response was more sensitive to heat stress than *C. cadmia*, by increasing the expression of photosynthesis-related genes in the early stage of heat stress. It increased the excitation energy transferred to the PSII core complex and promoted the increase of electron transfer efficiency. But continuous heat stress will lead to a decrease in the chlorophyll content and net photosynthetic rate.

In summary, these investigation results indicated *C. crassifolia* and *C. cadmia* exhibited different photosynthetic characteristic, metabolic characteristics, antioxidant system, and gene expression patterns, which were related to their suitable living environment and genetic evolution. And we found that the photosynthetic system and enzymatic system may be the key links in the response to heat stress of *C. crassifolia* and *C. cadmia*, respectively. The hypothesis will be tested in future work. This study will pave the way to research the response and tolerance molecular mechanisms in clematis under heat stress.

## Data Availability Statement

The original contributions presented in the study are included in the article/Supplementary Material, further inquiries can be directed to the corresponding author.

## Author Contributions

QH performed the experiments, analyzed the data, and completed the manuscript. RQ and YZ helped to perform the experiment. XZ and XM revised the manuscript. JZ approved the final version. All authors contributed to the article and approved the submitted version.

## Conflict of Interest

The authors declare that the research was conducted in the absence of any commercial or financial relationships that could be construed as a potential conflict of interest.

## Supplementary Material

Supplementary Figure 1 MDA content of *C. crassifolia* and *C. cadmia grown* under three different temperatures. Values are the means ± standard error (*n* = 5 plants). Different letters indicate significant differences based on two-way ANOVA followed by Tukey multiple comparisons (*P* ≤ 0.05).

Supplementary Table 1 The unigene annotations in the KEGG pathway database.

Supplementary Table 2 Primer sequences for gene expression analysis.

## References

[B1] Ahmad P. Jaleel C. A. Salem M. A. Nabi G. Sharma S. (2010). Roles of enzymatic and nonenzymatic antioxidants in plants during abiotic stress. *Crit. Rev. Biotechnol.* 30 161–175. 10.3109/07388550903524243 20214435

[B2] Alhaithloul H. A. Soliman M. H. Ameta K. L. El-Esawi M. A. Elkelish A. (2020). Changes in ecophysiology, osmolytes, and secondary metabolites of the medicinal plants of *Mentha piperita* and *Catharanthus roseus* subjected to drought and heat stress. *Biomolecules* 10:43. 10.3390/biom10010043 31892111 PMC7023420

[B3] Asada K. (2006). Production and scavenging of reactive oxygen species in chloroplasts and their functions. *Plant Physiol.* 141 391–396. 10.1104/pp.106.082040 16760493 PMC1475469

[B4] Atkinson N. J. Urwin P. E. (2012). The interaction of plant biotic and abiotic stresses: from genes to the field. *J. Exp. Bot.* 63 3523–3543. 10.1093/jxb/ers100 22467407

[B5] Baniwal S. K. Bharti K. Chan K. Y. Fauth M. Ganguli A. Kotak S. (2004). Heat stress response in plants: a complex game with chaperones and more than twenty heat stress transcription factors. *J. Biosci.* 29 471–487. 10.1007/BF02712120 15625403

[B6] Berry J. Bjorkman O. (1980). Photosynthetic response and adaptation to temperature in higher plants. *Ann. Rev. Plant Physiol.* 31 491–543. 10.1146/annurev.pp.31.060180.002423

[B7] Bickford C. P. (2016). Ecophysiology of leaf trichomes. *Funct. Plant Biol.* 43 807–814. 10.1071/FP16095 32480505

[B8] Bita C. Gerats T. (2013). Plant tolerance to high temperature in a changing environment: scientific fundamentals and production of heat stress-tolerant crops. *Front. Plant Sci.* 4:273. 10.3389/fpls.2013.00273 23914193 PMC3728475

[B9] Borrell J. S. Dodsworth S. Forest F. Pérez-Escobar O. A. Lee M. A. Mattana E. (2020). The climatic challenge: which plants will people use in the next century? *Environ. Exp. Bot.* 170:103872. 10.1016/j.envexpbot.2019.103872

[B10] Bowlus R. D. Somero G. N. (1979). Solute compatibility with enzyme function and structure: rationales for the selection of osmotic agents and end-products of anaerobic metabolism in marine invertebrates. *J. Exp. Zool.* 208 137–151. 10.1002/jez.1402080202 469480

[B11] Choudhury F. K. Rivero R. M. Blumwald E. Mittler R. (2017). Reactive oxygen species, abiotic stress and stress combination. *Plant J.* 90 856–867. 10.1111/tpj.13299 27801967

[B12] Crafts-Brandner S. J. Salvucci M. E. (2002). Sensitivity of photosynthesis in a C4 plant, maize, to heat stress. *Plant Physiol.* 129 1773–1780. 10.1104/pp.002170 12177490 PMC166765

[B13] Fan Y. Ma C. Huang Z. Abid M. Jiang S. Dai T. (2018). Heat priming during early reproductive stages enhances thermo-tolerance to post-anthesis heat stress via improving photosynthesis and plant productivity in winter wheat (*Triticum aestivum* L.). *Front. Plant Sci.* 9:805. 10.3389/fpls.2018.00805 29951079 PMC6008404

[B14] Gao L. Ma Y. Wang P. Wang S. A. Yang R. Wang Q. (2017). Transcriptome profiling of *Clematis apiifolia*: insights into heat-stress responses. *DNA Cell Biol.* 36 938–946. 10.1089/dna.2017.3850 28945464

[B15] Guo M. Liu J. H. Ma X. Luo D. X. Gong Z. H. Lu M. H. (2016). The plant heat stress transcription factors (HSFs): structure, regulation, and function in response to abiotic stresses. *Front. Plant Sci.* 7:114. 10.3389/fpls.2016.00114 26904076 PMC4746267

[B16] Gururani M. A. Venkatesh J. Tran L. S. P. (2015). Regulation of photosynthesis during abiotic stress-induced photoinhibition. *Mol. Plant* 8 1304–1320. 10.1016/j.molp.2015.05.005 25997389

[B17] Hameed A. Goher M. Iqbal N. (2012). Heat stress-induced cell death, changes in antioxidants, lipid peroxidation, and protease activity in wheat leaves. *J. Plant Growth Regulat.* 31 283–291. 10.1007/s00344-011-9238-4

[B18] Hao D. Gu X. Xiao P. Peng Y. (2013). Chemical and biological research of clematis medicinal resources. *Chinese Sci. Bull.* 58 1120–1129. 10.1007/s11434-012-5628-7

[B19] Hasanuzzaman M. Nahar K. Alam M. Roychowdhury R. Fujita M. (2013). Physiological, biochemical, and molecular mechanisms of heat stress tolerance in plants. *Int. J. Mol. Sci.* 14 9643–9684. 10.3390/ijms14059643 23644891 PMC3676804

[B20] Jacob P. Hirt H. Bendahmane A. (2017). The heat-shock protein/chaperone network and multiple stress resistance. *Plant Biotechnol. J.* 15 405–414. 10.1111/pbi.12659 27860233 PMC5362687

[B21] Jiang C. Bi Y. Mo J. Zhang R. Qu M. Feng S. (2020). Proteome and transcriptome reveal the involvement of heat shock proteins and antioxidant system in thermotolerance of *Clematis florida*. *Sci. Rep.* 10:8883. 10.1038/s41598-020-65699-2 32483281 PMC7264250

[B22] Kaplan F. Kopka J. Haskell D. W. Zhao W. Schiller K. C. Gatzke N. (2004). Exploring the temperature-stress metabolome of *Arabidopsis*. *Plant Physiol.* 136 4159–4168. 10.1104/pp.104.052142 15557093 PMC535846

[B23] Krasensky J. Jonak C. (2012). Drought, salt, and temperature stress-induced metabolic rearrangements and regulatory networks. *J. Exp. Bot.* 63 1593–1608. 10.1093/jxb/err460 22291134 PMC4359903

[B24] Larkindale J. Vierling E. (2008). Core genome responses involved in acclimation to high temperature. *PlantPhysiology* 146 748–761. 10.1104/pp.107.112060 18055584 PMC2245833

[B25] Lehtonen S. Christenhusz M. J. Falck D. (2016). Sensitive phylogenetics of Clematis and its position in ranunculaceae. *Botan. J. Linnean Soc.* 182 825–867. 10.1111/boj.12477

[B26] Li L. Ma Y. Gao L. Wang S. A. Wang P. Yang R. (2018). Association analysis of heat-resistance traits in *Clematis*. *Eur. J. Horticul. Sci.* 83 151–159. 10.17660/eJHS.2018/83.3.4

[B27] Li M. Zheng Y. Liang H. Zou L. Sun J. Zhang Y. (2013). Molecular cloning and characterization of cat, gpx1 and Cu/Zn-sod genes in pengze crucian carp (*Carassius auratus* var. Pengze) and antioxidant enzyme modulation induced by hexavalent chromium in juveniles. *Comparat. Biochem. Physiol. Part C: Toxicol. Pharmacol.* 157 310–321. 10.1016/j.cbpc.2013.02.003 23462276

[B28] Li R. Guo L. X. Li Y. Chang W. Q. Liu J. Q. Liu L. F. (2017). Dose-response characteristics of Clematis triterpenoid saponins and clematichinenoside AR in rheumatoid arthritis rats by liquid chromatography/mass spectrometry-based serum and urine metabolomics. *J. Pharm. Biomed. Anal.* 136 81–91. 10.1016/j.jpba.2016.12.037 28064091

[B29] Lichtenthaler H. K. Buschmann C. (2001). Chlorophylls and carotenoids: measurement and characterization by UV-VIS spectroscopy. *Curr. Protocols Food Anal. Chem.* 1 F4–F3. 10.1002/0471142913.faf0403s01

[B30] Liu G. Li W. Zheng P. Xu T. Chen L. Liu D. (2012). Transcriptomic analysis of ‘Suli’pear (*Pyrus pyrifolia* white pear group) buds during the dormancy by RNA-Seq. *BMC Genomics* 13:700. 10.1186/1471-2164-13-700 23234335 PMC3562153

[B31] Liu Z. Shao W. Shen Y. Ji M. Chen W. Ye Y. (2018). Characterization of new microsatellite markers based on the transcriptome sequencing of *Clematis finetiana*. *Hereditas* 155 1–9. 10.1186/s41065-018-0060-x 29785177 PMC5952850

[B32] Lohani N. Singh M. B. Bhalla P. L. (2020). High temperature susceptibility of sexual reproduction in crop plants. *J. Exp. Bot.* 71 555–568. 10.1093/jxb/erz426 31560053

[B33] Luo F. Lv Q. Zhao Y. Hu G. Huang G. Zhang J. (2012). Quantification and purification of mangiferin from Chinese mango (*Mangifera indica* L.) cultivars and its protective effect on human umbilical vein endothelial cells under H2O2-induced stress. *Int. J. Mol. Sci.* 13 11260–11274. 10.3390/ijms130911260 23109851 PMC3472743

[B34] Ma X. Zheng J. Zhang X. Hu Q. Qian R. (2017). Salicylic acid alleviates the adverse effects of salt stress on *Dianthus superbus* (Caryophyllaceae) by activating photosynthesis, protecting morphological structure, and enhancing the antioxidant system. *Front. Plant Sci.* 8:600. 10.3389/fpls.2017.00600 28484476 PMC5399920

[B35] Ma X. Qian R. Zhang X. Hu Q. Liu H. Zheng J. (2019). Contrasting growth, physiological and gene expression responses of *Clematis crassifolia* and *Clematis cadmia* to different irradiance conditions. *Scientific Reports* 9:17842. 10.1038/s41598-019-54428-z 31780789 PMC6883030

[B36] Mishra S. K. Tripp J. Winkelhaus S. Tschiersch B. Theres K. Nover L. (2002). In the complex family of heat stress transcription factors, HsfA1 has a unique role as master regulator of thermotolerance in tomato. *Genes Dev.* 16 1555–1567. 10.1101/gad.228802 12080093 PMC186353

[B37] Nayyar H. Kaur R. Kaur S. Singh R. (2014). γ-Aminobutyric acid (GABA) imparts partial protection from heat stress injury to rice seedlings by improving leaf turgor and upregulating osmoprotectants and antioxidants. *J. Plant Growth Regul.* 33 408–419. 10.1007/s00344-013-9389-6

[B38] Obokata J. Mikami K. Hayashida N. Nakamura M. Sugiura M. (1993). Molecular heterogeneity of photosystem I (psaD, psaE, psaF, psaH, and psaL are all present in isoforms in Nicotiana spp.). *Plant Physiol.* 102 1259–1267. 10.1104/pp.102.4.1259 8278548 PMC158913

[B39] Ohama N. Kusakabe K. Mizoi J. Zhao H. Kidokoro S. Koizumi S. (2016). The transcriptional cascade in the heat stress response of *Arabidopsis* is strictly regulated at the level of transcription factor expression. *Plant Cell* 28 181–201. 10.1105/tpc.15.00435 26715648 PMC4746676

[B40] Ohama N. Sato H. Shinozaki K. Yamaguchi-Shinozaki K. (2017). Transcriptional regulatory network of plant heat stress response. *Trends Plant Sci.* 22 53–65. 10.1016/j.tplants.2016.08.015 27666516

[B41] Ouyang S. Q. Liu Y. F. Liu P. Lei G. He S. J. Ma B. (2010). Receptor-like kinase OsSIK1 improves drought and salt stress tolerance in rice (*Oryza sativa*) plants. *Plant J.* 62 316–329. 10.1111/j.1365-313X.2010.04146.x 20128882

[B42] Ozawa S. I. Bald T. Onishi T. Xue H. Matsumura T. Kubo R. (2018). Configuration of ten light-harvesting chlorophyll a/b complex I subunits in Chlamydomonas reinhardtii photosystem I. *Plant Physiol.* 178 583–595. 10.1104/pp.18.00749 30126869 PMC6181050

[B43] Pnueli L. Liang H. Rozenberg M. Mittler R. (2003). Growth suppression, altered stomatal responses, and augmented induction of heat shock proteins in cytosolic ascorbate peroxidase (Apx1)-deficient *Arabidopsis* plants. *Plant J.* 34 187–203. 10.1046/j.1365-313X.2003.01715.x 12694594

[B44] Pringle J. S. (1971). Taxonomy and distribution of Clematis, sect. Atragene (Ranunculaceae), in North America. *Brittonia* 23 361–393. 10.2307/2805705

[B45] Priya M. Sharma L. Singh I. Bains T. S. Siddique K. H. Bindumadhava H. (2019). Securing reproductive function in mungbean grown under high temperature environment with exogenous application of proline. *Plant Physiol. Biochem.* 140 136–150. 10.1016/j.plaphy.2019.05.009 31103796

[B46] Qu A. L. Ding Y. F. Jiang Q. Zhu C. (2013). Molecular mechanisms of the plant heat stress response. *Biochem. Biophys. Res. Commun.* 432 203–207. 10.1016/j.bbrc.2013.01.104 23395681

[B47] Rizhsky L. Liang H. Shuman J. Shulaev V. Davletova S. Mittler R. (2004). When defense pathways collide. The response of *Arabidopsis* to a combination of drought and heat stress. *Plant Physiol.* 134 1683–1696. 10.1104/pp.103.033431 15047901 PMC419842

[B48] Rodríguez M. Canales E. Borrás-Hidalgo O. (2005). Molecular aspects of abiotic stress in plants. *Biotecnol. Aplicada* 22 1–10.

[B49] Rodríguez R. J. Henson J. Van Volkenburgh E. Hoy M. Wright L. Beckwith F. (2008). Stress tolerance in plants via habitat-adapted symbiosis. *ISME J.* 2 404–416. 10.1038/ismej.2007.106 18256707

[B50] Röth S. Mirus O. Bublak D. Scharf K. D. Schleiff E. (2017). DNA-binding and repressor function are prerequisites for the turnover of the tomato heat stress transcription factor HsfB1. *Plant J.* 89 31–44. 10.1111/tpj.13317 27560701

[B51] Shi Q. Bao Z. Zhu Z. Ying Q. Qian Q. (2006). Effects of different treatments of salicylic acid on heat tolerance, chlorophyll fluorescence, and antioxidant enzyme activity in seedlings of Cucumis sativa L. *Plant Growth Regulat.* 48 127–135. 10.1007/s10725-005-5482-6

[B52] Sita K. Sehgal A. Bhandari K. Kumar J. Kumar S. Singh S. (2018). Impact of heat stress during seed filling on seed quality and seed yield in lentil (*Lens culinaris* Medikus) genotypes. *J. Sci. Food Agricul.* 98 5134–5141. 10.1002/jsfa.9054 29635707

[B53] Tozzi E. S. Easlon H. M. Richards J. H. (2013). Interactive effects of water, light and heat stress on photosynthesis in F remont cottonwood. *Plant, Cell Environ.* 36 1423–1434. 10.1111/pce.12070 23336343

[B54] Tripathy B. C. Oelmüller R. (2012). Reactive oxygen species generation and signaling in plants. *Plant Signal. Behav.* 7 1621–1633. 10.4161/psb.22455 23072988 PMC3578903

[B55] Vidya M. K. Kumar V. G. Sejian V. Bagath M. Krishnan G. Bhatta R. (2018). Toll-like receptors: significance, ligands, signaling pathways, and functions in mammals. *Int. Rev. Immunol.* 37 20–36. 10.1080/08830185.2017.1380200 29028369

[B56] Wahid A. Gelani S. Ashraf M. Foolad M. R. (2007). Heat tolerance in plants: an overview. *Environ. Exp. Bot.* 61 199–223. 10.1016/j.envexpbot.2007.05.011

[B57] Wang X. Huang W. Liu J. Yang Z. Huang B. (2017). Molecular regulation and physiological functions of a novel FaHsfA2c cloned from tall fescue conferring plant tolerance to heat stress. *Plant Biotechnol. J.* 15 237–248. 10.1111/pbi.12609 27500592 PMC5258862

[B58] Wise R. R. Olson A. J. Schrader S. M. Sharkey T. D. (2004). Electron transport is the functional limitation of photosynthesis in field-grown Pima cotton plants at high temperature. *Plant, Cell Environ.* 27 717–724. 10.1111/j.1365-3040.2004.01171.x

[B59] Wu H. C. Bulgakov V. P. Jinn T. L. (2018). Pectin methylesterases: cell wall remodeling proteins are required for plant response to heat stress. *Front. Plant Sci.* 9:1612. 10.3389/fpls.2018.01612 30459794 PMC6232315

[B60] Xu W. Cai S. Y. Zhang Y. Wang Y. Ahammed G. J. Xia X. J. (2016). Melatonin enhances thermotolerance by promoting cellular protein protection in tomato plants. *J. Pineal Res.* 61 457–469. 10.1111/jpi.12359 27484733

[B61] Yin H. Chen Q. Yi M. (2008). Effects of short-term heat stress on oxidative damage and responses of antioxidant system in *Lilium longiflorum*. *Plant Growth Regulat.* 54 45–54. 10.1007/s10725-007-9227-6

[B62] Yoshida T. Ohama N. Nakajima J. Kidokoro S. Mizoi J. Nakashima K. (2011). Arabidopsis HsfA1 transcription factors function as the main positive regulators in heat shock-responsive gene expression. *Mol. Genet. Genom.* 286 321–332. 10.1007/s00438-011-0647-7 21931939

[B63] Zheng J. Ma X. Zhang X. Hu Q. Qian R. (2018). Salicylic acid promotes plant growth and salt-related gene expression in *Dianthus superbus* L. (Caryophyllaceae) grown under different salt stress conditions. *Physiol. Mol. Biol. Plants* 24 231–238. 10.1007/s12298-017-0496-x 29515317 PMC5834982

[B64] Zhou J. Xu X. C. Cao J. J. Yin L. L. Xia X. J. Shi K. (2018). Heat shock factor HsfA1a Is essential for R Gene-Mediated nematode resistance and triggers H2O2 Production1. *Plant Physiol.* 176 2456–2471. 10.1104/pp.17.01281 29339397 PMC5841716

[B65] Zhou R. Kong L. Yu X. Ottosen C. O. Zhao T. Jiang F. (2019). Oxidative damage and antioxidant mechanism in tomatoes responding to drought and heat stress. *Acta Physiol. Plantarum* 41:20. 10.1007/s11738-019-2805-1

[B66] Zhou W. Leul M. (1999). Uniconazole-induced tolerance of rape plants to heat stress in relation to changes in hormonal levels, enzyme activities and lipid peroxidation. *Plant Growth Regulat.* 27 99–104. 10.1023/A:1006165603300

