# Supplementary figures and images for: Physiological and Gene Expression Changes of Clematis crassifolia and Clematis cadmia in Response to Heat Stress
Source: Front Plant Sci. 2021 Mar 26;12:624875. doi: 10.3389/fpls.2021.624875 (PMC8034387; doi:10.3389/fpls.2021.624875)

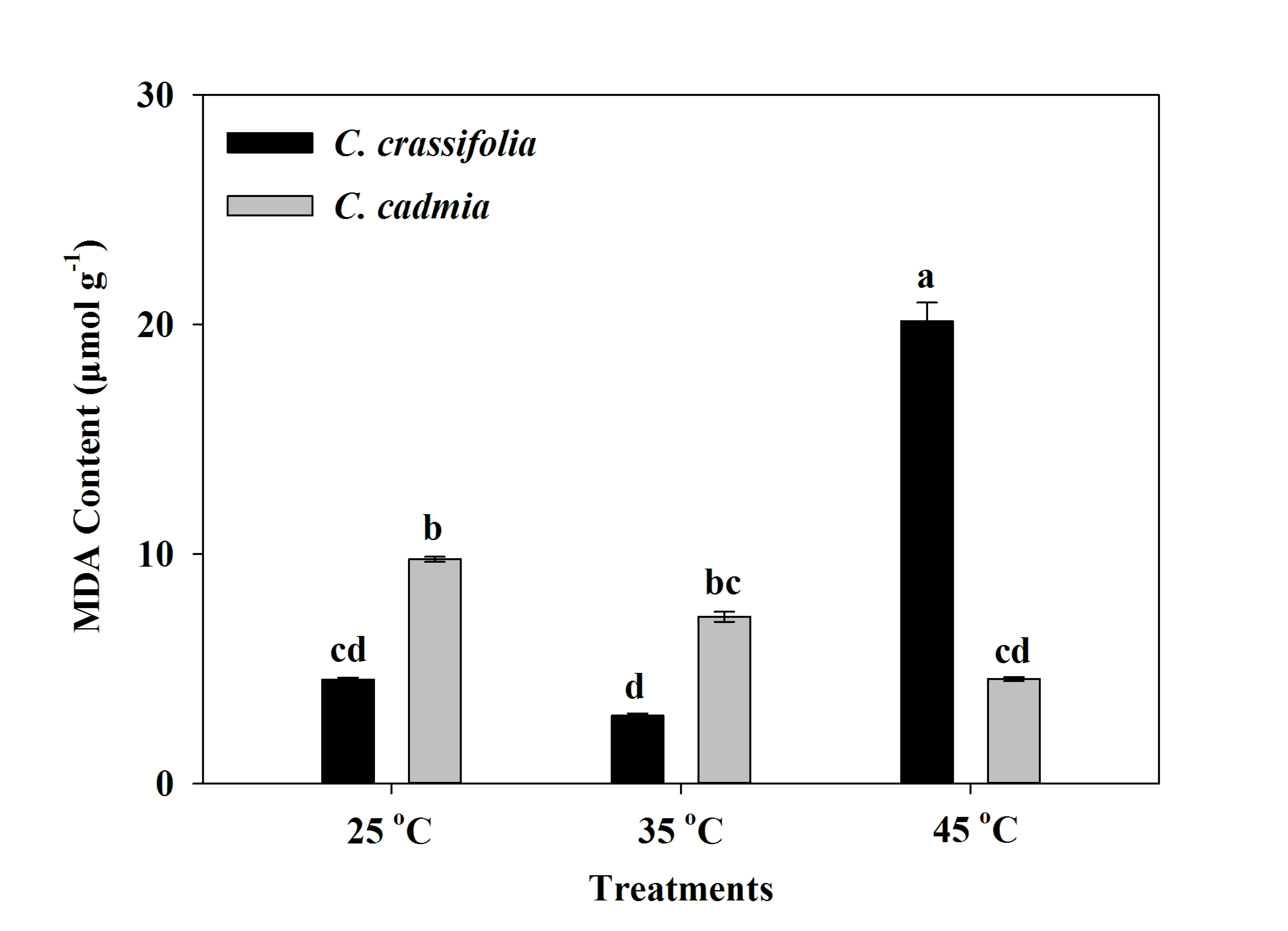

Supplement: Supplementary Figure 1 — MDA content of C. crassifolia and C. cadmia grown under three different temperatures. Values are the means ± standard error (n = 5 plants). Different letters indicate significant differences based on two-way ANOVA followed by Tukey multiple comparisons (P ≤ 0.05). [file Image_1.TIF]
