# Supplementary material for: Physiological and Gene Expression Changes of Clematis crassifolia and Clematis cadmia in Response to Heat Stress
Source: Front Plant Sci. 2021 Mar 26;12:624875. doi: 10.3389/fpls.2021.624875 (PMC8034387; doi:10.3389/fpls.2021.624875)
Supplement: Supplementary Table 1 — The unigene annotations in the KEGG pathway database. [file Table_1.DOCX]

**Table S1.** The unigene annotations in the KEGG pathway database.

| Genes code | Gene ID | Description |
| --- | --- | --- |
| c194329_g3 | gi\|1012343194 | 17.4 kda class III heat shock protein |
| c204515_g1 | gi\|720090738 | Heat shock transcription factor A1 |
| c194434_g1 | gi\|720073097 | Heat shock transcription factor B-2b-like |
| c188817_g1 | gi\|719980139 | Photosystem I reaction center subunit VI-2 |
| c208712_g3 | gi\|944542068 | Photosystem II protein D |
| c200811_g3 | gi\|224110818 | Photosystem II core complex proteins psbY |
| c187075_g1 | gi\|77157635 | Chlorophyll a/b binding protein |
| c194962_g2 | gi\|959993683 | Chlorophyll a/b-binding protein1 |
| c199977_g2 | gi\|816206858 | Peroxidase |
| c202620_g2 | gi\|729420607 | Superoxide dismutase 1 |
| c195983_g1 | gi\|697128179 | L-ascorbate peroxidase 3 |
| c198009_g1 | gi\|75127599 | Peroxiredoxin Q |
