# Supplementary material for: Physiological and Gene Expression Changes of Clematis crassifolia and Clematis cadmia in Response to Heat Stress
Source: Front Plant Sci. 2021 Mar 26;12:624875. doi: 10.3389/fpls.2021.624875 (PMC8034387; doi:10.3389/fpls.2021.624875)
Supplement: Supplementary Table 2 — Primer sequences for gene expression analysis. [file Table_2.DOCX]

**Table S2.** Primer sequence for gene expression analysis of *C. crassifolia*.

| Gene | Primer | Sequence 5’-3’ | Products scizes (bp) | Annealing temp (°C) |
| --- | --- | --- | --- | --- |
| Actin | Actin-F | AACCCTGAGGAGATTCCA | 60 | 162 |
|  | Actin-R | CACCACCCTTCAAGTGAGCAG |  |  |
| c194329_g3 | c194329_g3-F | TGAGGAGCAATGGGAAGAGG | 181 | 87.0 |
|  | c194329_g3-R | TGCTTCTCCACGGTAACACT |  |  |
| c204515_g1 | c204515_g1-F | GCCAATGTCAGGGTTGTCAA | 194 | 87.0 |
|  | c204515_g1-R | CCGCTACTTGAGGCAATTCC |  |  |
| c194434_g1 | c194434_g1-F | ACCAATCCAGACTGCCTCTC | 204 | 88.8 |
|  | c194434_g1-R | TCTCTTCTCAGCCGTTCGTT |  |  |
| c188817_g1 | c188817_g1-F | TTGGAAGACATCGGCAACAC | 164 | 86.1 |
|  | c188817_g1-R | GAAGTAGGCAAGAGTGGAGC |  |  |
| c208712_g3 | c208712_g3-F | GGGTCGTGAGTGGGAACTTA | 191 | 87.1 |
|  | c208712_g3-R | TGTGCTCAGCCTGGAATACA |  |  |
| c200811_g3 | c200811_g3-F | ATCGTCCCGGCTATTCTCTG | 198 | 87.2 |
|  | c200811_g3-R | ACCCCTGCTATCATTCGAGG |  |  |
| c187075_g1 | c187075_g1-F | TGGGCTTTGTTGAGGGATAC | 169 | 89.7 |
|  | c187075_g1-R | ATTGAGAACATTGCGAGCCG |  |  |
| c194962_g2 | c194962_g2-F | GGAACTCAAGAATGGACGGC | 156 | 84.8 |
|  | c194962_g2-R | CCGGGAACAAAGTTAGTGGC |  |  |
| c199977_g2 | c199977_g2-F | CATCTTCCATGACTGTGCCG | 171 | 87.2 |
|  | c199977_g2-R | CAAGAAACAACACCAGGGCA |  |  |
| c202620_g2 | c202620_g2-F | ACTCTCCCCGATCTCCCATA | 181 | 87.3 |
|  | c202620_g2-R | CGCTCTGCAACTTGACAACA |  |  |
| c195983_g1 | c195983_g1-F | TCTCCGTGCTCTCATTTCCA | 155 | 85.7 |
|  | c195983_g1-R | AGCCATTGTTTGCACCATGT |  |  |
| c198009_g1 | c198009_g1-F | AGACCCCTGGCTGTACTAAAC | 140 | 84.6 |
|  | c198009_g1-R | TGGAAGCTTGTATTTCTTGGCA |  |  |
